# The Selective Impairment of Resting-State Functional Connectivity of the Lateral Subregion of the Frontal Pole in Schizophrenia

**DOI:** 10.1371/journal.pone.0119176

**Published:** 2015-03-06

**Authors:** Yujing Zhou, Xiaomei Ma, Di Wang, Wen Qin, Jiajia Zhu, Chuanjun Zhuo, Chunshui Yu

**Affiliations:** 1 Department of Radiology and Tianjin Key Laboratory of Functional Imaging, Tianjin Medical University General Hospital, Tianjin, China; 2 Department of Psychiatry Functional Neuroimaging Laboratory, Tianjin Mental Health Center, Tianjin Anding Hospital, Tianjin, China, and Tianjin Anning Hospital, Tianjin, China; & National Laboratory of Pattern Recognition, CHINA

## Abstract

**Objective:**

Although extensive resting-state functional connectivity (rsFC) changes have been reported in schizophrenia, rsFC changes of the frontal pole (FP) remain unclear. The FP contains several subregions with different connection patterns; however, it is unknown whether the FP subregions are differentially affected in schizophrenia. To explore this possibility, we compared rsFC differences of the FP subregions between schizophrenia patients and healthy controls.

**Method:**

One hundred healthy controls and 91 patients with schizophrenia underwent resting-state functional MRI with a sensitivity-encoded spiral-in (SENSE-SPIRAL) imaging sequence to reduced susceptibility-induced signal loss and distortion. The FP was subdivided into the orbital (FPo), medial (FPm), and lateral (FPl) subregions. Mean fMRI time series were extracted for each FP subregion and entered into a seed-based rsFC analysis.

**Results:**

The FP subregions exhibited differential rsFC patterns in both healthy controls and schizophrenia patients. Direct comparison between groups revealed reduced rsFCs between the bilateral FPl and several cognitive-related regions, including the dorsolateral prefrontal cortex, medial prefrontal cortex, anterior cingulate cortex, posterior cingulate cortex/precuneus, temporal cortex and inferior parietal lobule in schizophrenia. Although the FPl exhibited obvious atrophy, rsFC changes were unrelated to volumetric atrophy in the FPl, to duration of illness, and to antipsychotic medication dosage. No significant differences were observed in the rsFCs of other FP subregions.

**Conclusion:**

These findings suggest a selective (the lateral subregion) functional disconnection of the FP subregions in schizophrenia.

## Introduction

Schizophrenia is a severe and disabled psychiatric disorder and impairs multiple aspects of brain function, including perception, cognition and emotion [1]. Most of these functions depend on structural and functional integrity of the prefrontal cortex (PFC). The frontal pole (FP) locates in the anterior part of the PFC and experiences highly evolutionary expansion in humans [2, 3]. On the basis of neuroimaging studies, the human FP is thought to play a role in processing social and emotional information [4, 5], complex cognition [6–10] and self-referential tasks [11, 12], all of which are impaired in schizophrenia. Using task-based functional imaging methods, abnormal activation of the FP has been repeatedly found in schizophrenia [13–17]. A recent study points out that the FP network may play an important role in the pathophysiology of schizophrenia [18]. Moreover, structural atrophy of the FP has not only been found in schizophrenia patients but also in young relatives at risk for schizophrenia [19–21].

The disconnection hypothesis of schizophrenia proposes a reduced capacity to integrate information between distinct brain regions [22]. This hypothesis has been confirmed in multiple investigations and is considered as an important hallmark of schizophrenia. However, the connectivity changes of the FP have not yet been explored in schizophrenia patients. The human FP is not a functionally homogeneous region, suggesting the presence of subregions. Indeed, a recent MRI-based parcellation study has subdivided the FP into the orbital (FPo), medial (FPm), and lateral (FPl) subregions [2]. The authors further confirm that the FPo mainly connects with brain regions involving in socio-emotional processing, the FPm mainly connects with brain regions involving in self-referential processing, and the FPl mainly connects with brain regions involving in cognitive processing. This study suggests that investigation of the connectivity changes of the FP subregions may improve our understanding of the role of the FP in schizophrenia.

One of the most popular methods for studying connectivity is the resting-state functional connectivity (rsFC) that characterizes statistical dependency of functional MRI (fMRI) signals between two brain regions. Most previous rsFC studies have used echo-planar imaging (EPI) technique, which is challenged by susceptibility-induced signal loss and distortion in brain regions near air/tissue interfaces. To reduce susceptibility artifacts in these regions, a sensitivity-encoded spiral (SENSE-SPIRAL) imaging technique has been proposed to acquire fMRI data [23]. This technique may improve the fMRI quality of the FP and make rsFC analysis of the FP more reliable than the conventional EPI sequence.

In this study, we applied the SENSE-SPIRAL technique to acquire fMRI data and compared rsFC differences of each FP subregion between schizophrenia patients and healthy controls. We aimed to identify specific change patterns of each FP subregion in schizophrenia.

## Materials and Methods

### Participants

A total of 98 patients with schizophrenia and 102 healthy controls were recruited for this study. Diagnoses for patients were confirmed using the Structured Clinical Interview for DSM-IV. Inclusion criteria were age (18–55 years) and right-handedness. Exclusion criteria were MRI contraindications, poor image quality, presence of a systemic medical illness or CNS disorder, history of head trauma, and substance abuse within the last 3 months or lifetime history of substance abuse or dependence. Additional exclusion criteria for healthy comparison subjects were history of any Axis I or II disorders and a psychotic disorder and first-degree relative with a psychotic disorder. Four schizophrenia patients were excluded because of their oversized head motion (translational or rotational motion parameters more than 2 mm or 2°). After excluding subjects with poor image quality of the FP, 91 schizophrenia patients and 100 healthy controls were finally included. Clinical symptoms of psychosis were quantified with the Positive and Negative Syndrome Scale (PANSS) [24]. This study was approved by the Medical Research Ethics Committee of Tianjin Medical University General Hospital, and all participants provided written informed consent.

### Image data acquisition

MRI was performed using a 3.0-Tesla MR system (Discovery MR750, General Electric, Milwaukee, WI, USA). Tight but comfortable foam padding was used to minimize head motion, and earplugs were used to reduce scanner noise. Sagittal 3D T1-weighted images were acquired by a brain volume sequence with the following scan parameters: repetition time (TR) = 8.2 ms; echo time (TE) = 3.2 ms; inversion time (TI) = 450 ms; flip angle (FA) = 12°; field of view (FOV) = 256 mm × 256 mm; matrix = 256 × 256; slice thickness = 1 mm, no gap; and 188 sagittal slices. Two sets of the resting-state fMRI data were acquired. A gradient-echo single-short EPI sequence was performed using the following parameters: TR/TE = 2000/45 ms; FOV = 220 mm × 220 mm; matrix = 64 × 64; FA = 90°; slice thickness = 4 mm; gap = 0.5 mm; 32 interleaved transverse slices; 180 volumes. A gradient-echo SENSE-SPIRAL (spiral in) sequence was performed using parameters of TR/TE = 1400/30 ms; FA = 60°, acceleration factor = 2. The FOV, matrix, slice thickness, gap, and slice number were the same as the EPI sequence. During fMRI scans, all subjects were instructed to keep their eyes closed, to relax and move as little as possible, to think of nothing in particular, and to not fall asleep.

### Gray matter volume (GMV) calculation

The GMV of each voxel was calculated using Statistical Parametric Mapping software (SPM8; http://www.fil.ion.ucl.ac.uk/spm/software/spm8/). The structural MR images were segmented into gray matter (GM), white matter and cerebrospinal fluid using the standard unified segmentation model. After an initial affine registration of GM concentration map into the Montreal Neurological Institute (MNI) space, GM concentration images were nonlinearly warped using diffeomorphic anatomical registration through the exponentiated Lie algebra (DARTEL) technique and were resampled to 1.5-mm cubic voxels. The GMV of each voxel was obtained by multiplying GM concentration map by the non-linear determinants derived from the spatial normalization step. Finally, GMV images were smoothed with a Gaussian kernel of 6 × 6 × 6 mm^3^ full-width at half maximum (FWHM). After spatial preprocessing, the normalized, modulated, and smoothed GMV maps were used for statistical analysis.

### Data preprocessing

Two sets of resting-state fMRI data were preprocessed using the SPM8 with the same procedures. The first 10 volumes for each subject were discarded to allow the signal to reach equilibrium and the participants to adapt to the scanning noise. The remaining volumes were then corrected for the acquisition time delay between slices. All subjects’ fMRI data were within defined motion thresholds (translational or rotational motion parameters less than 2 mm or 2°). We also calculated framewise displacement (FD), which indexes volume-to-volume changes in head position [25]. Considering recent studies reported that signal spike caused by head motion significantly contaminated the final resting-state fMRI results even after regressing out the realignment parameters [25], we removed spike volumes when the FD of specific volume exceeded 0.5. Several nuisance covariates (six motion parameters and average BOLD signals of the ventricular, white matter and whole brain) were regressed out from the data. The datasets were band-pass filtered with a frequency range of 0.01 to 0.08 Hz. Individual structural images were linearly coregistered to the mean functional image; then the transformed structural images were segmented into GM, white matter, and cerebrospinal fluid. The GM maps were linearly coregistered to the tissue probability maps in the MNI space. Finally the motion-corrected functional volumes were spatially normalized to the MNI space using the parameters estimated during linear coregistration. The functional images were resampled into 3 × 3 × 3 mm^3^ voxels. After normalization, all datasets were smoothed with a Gaussian kernel of 6 × 6 × 6 mm^3^ FWHM.

### Definition of the FP subregions

The bilateral FP subregions were defined according to the maximal probability maps from a previous parcellation study of the FP [2], which the FP was parcellated into the FPo, FPm and FPl subregions. Thus, we defined a total of 6 regions of interest (ROIs), including the FPo, FPm and FPl bilaterally.

### Signal intensity assessments

Conventional EPI images of the FP are apt to susceptibility-induced signal loss and distortion; however, the SENSE-SPIRAL sequence may at least partly reduce the effects. We first compared the signal intensity and distortion of the FP derived from the two resting-state fMRI sequences. The relative signal intensity (rSI) of each FP region was calculated by dividing the mean signal intensity of the whole brain gray matter and was compared between the two imaging methods. The distortion severity was assessed by observing deviations of the normalized functional images from the structural images. As expected, the SENSE-SPIRAL sequence had a better image quality, and then the fMRI data from this sequence were used to perform rsFC analysis. To exclude possible effect of signal loss on our results, we excluded subjects whose rSI of any FP subregion was lower than 0.5.

### rsFC analysis

For individual dataset, Pearson’s correlation coefficients between the mean time series of each ROI and time series of each voxel in other parts of the brain GM were computed, and converted to *z* values using Fisher’s *r-*to-*z* transformation to improve the normality. Individuals’ *z* values were then entered into a random-effect one-sample *t*-test in a voxel-wise manner to identify brain regions that showed significant positive correlations with each ROI. Multiple comparisons for this analyses were corrected for using a family wise error (FWE) method (*p*<0.05).Then a two-sample *t*-test was performed within the positive rsFC mask to quantitatively compare group differences in rsFCs of the 6 ROIs after controlling for age and gender. Multiple comparisons for this analyses were corrected for using a false positive rate (FDR) method (*p*<0.05). To exclude the effect of GMV on rsFC changes, we extracted the GMV of the FP subregions and re-evaluated the rsFC changes of each FP subregion after further controlling for the GMV of the FP subregion.

### Validation analysis

Considering that ROIs extracted from the maximal probability maps may result in information overlap across ROIs due to the preprocessing steps of normalization and smoothing, we also defined these ROIs using an alternative method (spheres with a radius of 6 mm centered at the gravity of each FP subregion) to exclude the effect. We repeated our rsFC analyses to test whether different methods for ROI definition influence our results.

### Correlations between rsFCs of the FP subregions and clinical parameters

To further test whether the rsFCs of the FP subregions with significant group differences were correlated with the clinical variables, we extracted the rsFCs of the FP subregions that exhibited significant group differences, and calculated Spearman’s correlation coefficients between these rsFCs and clinical parameters (i.e., PANSS, duration of the illness, and antipsychotic dosage) (*p*<0.05).

## Results

### Assessments of signal intensity and distortion of the FP subregions

**Fig 1:**
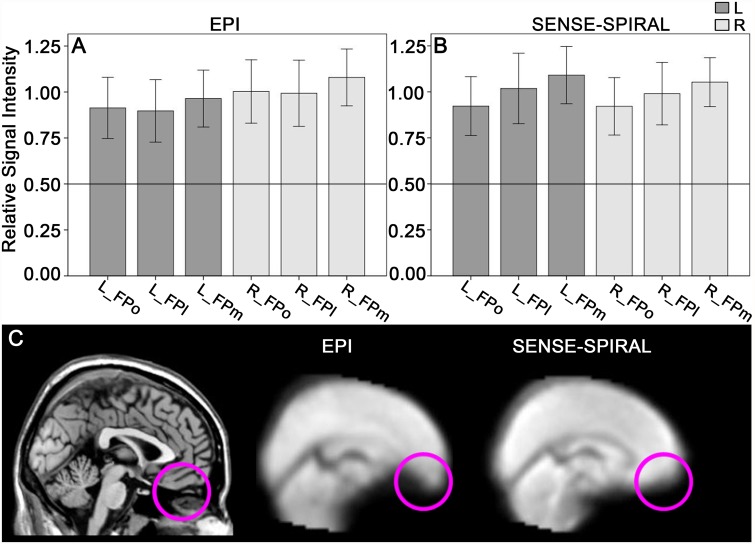
Assessments of signal intensity and distortion of the FP subregions. The mean rSI of the FP subregions derived from the echo planar imaging (EPI) sequence (A) and the sensitivity-encoded spiral-in imaging (SENSE-SPIRAL) sequence (B) are both higher than 0.5. The horizontal line indicates that the rSI is 0.5. Sagittal functional images (C) show that the frontal pole and orbitofrontal cortex exhibit less distortion in fMRI data acquired by the SENSE-SPIRAL sequence than in those acquired by the EPI sequence. rSI, the mean relative signal intensity; FPo, orbital subregion of the frontal pole; FPm, medial subregion of the frontal pole; FPl, lateral subregion of the frontal pole; L, left; R, right.

The mean rSI of each FP ROI is shown in Fig. 1. Although the mean rSIs of all FP ROIs derived from the SENSE-SPIRAL and the EPI fMRI were both higher than 0.5, but the normalized functional images derived from the SENSE-SPIRAL fMRI exhibited a less distortion in the FP and orbitofrontal cortex than those derived from the EPI fMRI (Fig. 1). Therefore, the functional images derived from the SENSE-SPIRAL fMRI were used for the rsFC analysis. Moreover, we excluded subjects whose rSI of any region was lower than 0.5 to ensure the signal intensity of the FP was large enough. This procedure excluded 3 patients and 2 healthy comparison subjects.

### Demographic and clinical characteristics of subjects

We finally included 91 schizophrenia patients (51 males; age: 33.8 ± 7.7 years) and 100 healthy controls (45 males; age: 33.3 ± 10.5 years). The demographic and clinical characteristics of these subjects are summarized in Table 1. There were no significant group differences in sex (χ^2^ = 2.324, df = 1, *p* = 0.127) and age (t = -0.341, df = 189, *p* = 0.734). To further exclude effects of sex and age on our results, we considered these two variables as covariates of no interest throughout rsFC analyses. Eighty-five patients were receiving medications when performing the MRI examinations and the other 6 patients have never received any medications.

**Table 1:** Demographic and clinical characteristics of schizophrenias and healthy comparison subjects.

Characteristic	Healthy Comparison Subjects (N = 100)		Schizophrenia Patients (N = 91)		Analysis		
	Mean	SD	Mean	SD	t/χ^2^	df	*p*
Age(years)	33.3	10.5	33.8	7.7	t = -0.34	189	0.73
Sex	M45	F55	M51	F40	χ^2^ = 2.32	1	0.13
Duration of illness (months)			120.1	90.7			
PANSS positive score			16.6	8.0			
PANSS negative score			20.0	8.4			
Current antipsychotic dosage (chlorpromazine equivalents) (mg/d)			447.4	338.8			

PANSS indicates Positive and Negative Syndrome Scale.

### The rsFC map of each group

**Fig 2:**
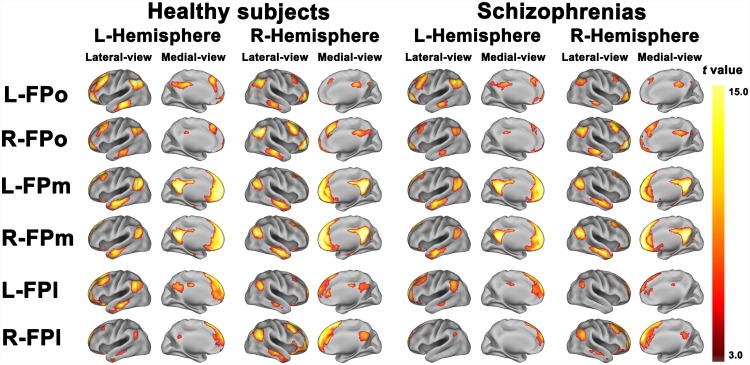
The resting-state functional connectivity map of each subregion of the frontal pole of each group.

The rsFC map of each FP subregion in each group is depicted in Fig. 2. Overall, in healthy controls the three FP subregions not only showed different rsFCs, representing functional segregation, but also displayed similar rsFCs, reflecting functional integration. Consistent with the previous study [2], the FPo was mainly correlated with the orbital frontal cortex (OFC), the FPl was mainly correlated with the dorsolateral prefrontal cortex (DLPFC), and the FPm was correlated with the brain regions belonged to the default-mode network (DMN), including the medial prefrontal cortex (MPFC), anterior cingulate cortex (ACC), posterior cingulate cortex/precuneus (PCC/Pcu), temporal cortex (TC), and inferior parietal lobule (IPL). Moreover, different FP subregions exhibited similar correlation with the MPFC, TC, PCC/Pcu, and IPL. Compared with healthy controls, schizophrenia patients showed similar rsFC patterns but with a less spatial extent.

Only positive resting-state functional connectivity map of each subregion of the frontal pole of each group is depicted. Images were thresholded with a FWE correction, p<0.05, two-tailed, cluster size>30 voxels. FPo, orbital subregion of the frontal pole; FPm, medial subregion of the frontal pole; FPl, lateral subregion of the frontal pole; L, left; R, right.

### The rsFC differences of the FP subregions between groups

**Fig 3:**
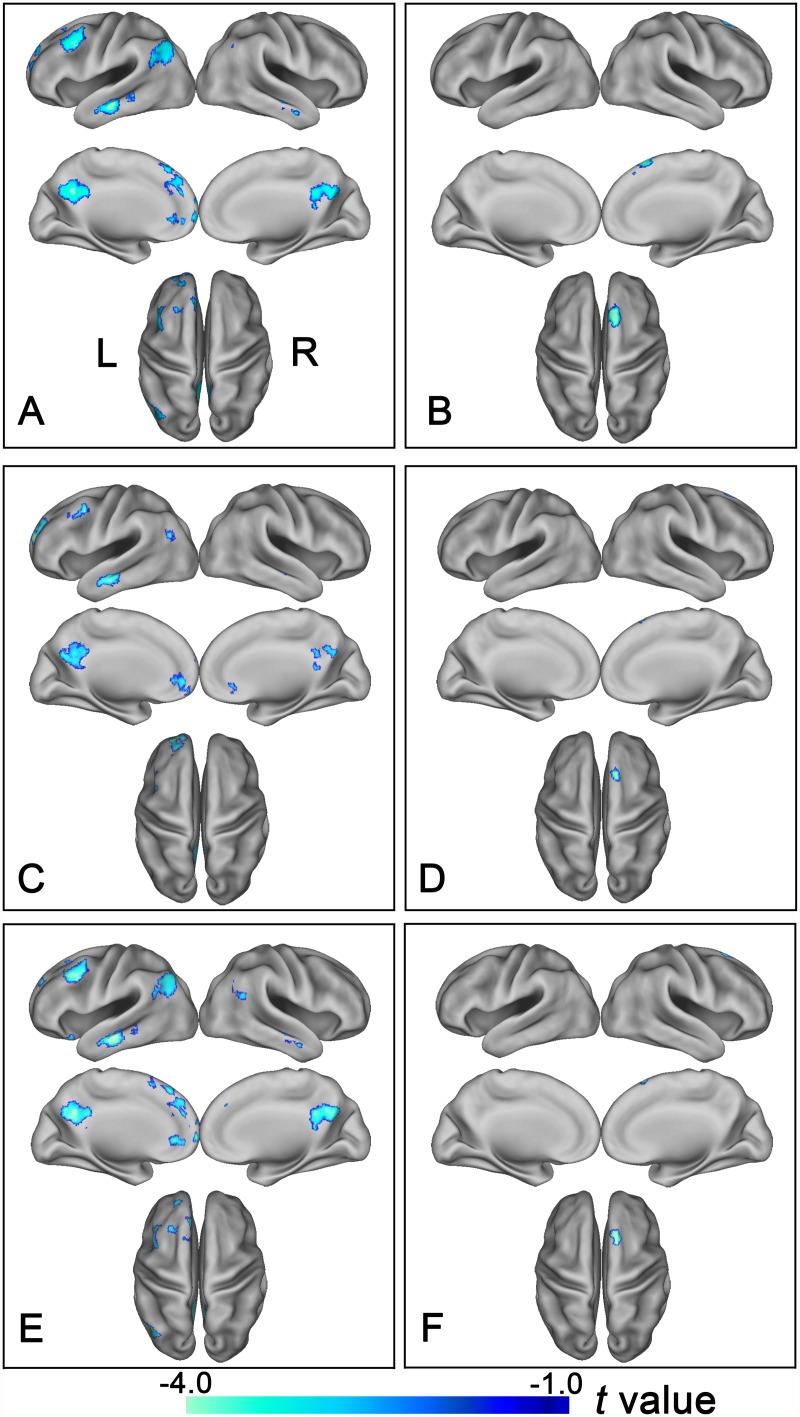
Altered rsFC of the bilateral lateral subregions of the frontal pole in schizophrenia patients. Brain regions exhibit significantly reduced rsFC with the left FPl (A, C) and the right FPl (B, D) in schizophrenia patients. (A, B) show results derived from the method of the maximal probability map and (C, D) demonstrate results derived from the method that using spherical regions of interest as seed regions. Of note, the between-group differences in the rsFC of the bilateral lateral subregions of the FP exhibited highly similar patterns between with (E and F) and without (A, B) correcting GM volume. The statistical threshold was set at p<0.05, FDR correction, two-tailed and cluster size>30 voxels. FPl, lateral subregion of the frontal pole; FP, frontal pole; L, left, R, right.

Group differences in rsFCs of the FP subregions are summarized in Table 2, Fig. 3A and B. Compared with healthy controls, schizophrenia patients exhibited reduced rsFCs between the left FPl and brain regions that are involved in cognitive processing, including the left DLPFC, ACC, MPFC, TC, PCC/Pcu, and IPL. The patients also had reduced rsFCs between the right FPl and the right superior frontal gyrus (SFG) (*p*<0.05, two-tailed, FDR correction, cluster size >30 voxels). To further determine the disconnection pattern of the right FPl, we used an uncorrected threshold of p<0.05 and found that the disconnection pattern of the right FPl was similar to that of the left FPl (S1 Fig). No group differences were observed in the bilateral FPo and the bilateral FPm.

**Table 2:** Brain regions with significant differences in resting-state functional connectivity of the frontal pole subregions between schizophrenia patients and healthy controls.

ROI	Brain Regions ^a^	Montreal Neurological Coordinates (x,y,z)	Peak T Values	Cluster Size (Voxels)^b^
Left FPl	Left Middle Temporal Gyrus	-51, -18,-21	-5.15	243
	Right Middle Temporal Gyrus	63,-15,-9	-4.48	68
	Left Anterior cingulate cortex	-3,42,-3	-3.88	65
	Left Superior Frontal Gyrus	-24,60,18	-3.69	145
	Left Medial Superior Frontal Gyrus	0,36,48	-3.80	153
	Left Middle Frontal Gyrus	-39,12,39	-4.18	226
	Left Precuneus	-12-57 30	-4.83	371
	Left Angular Gyrus	-45,-69,45	-3.77	220
	Right Angular Gyrus	57,-63,39	-3.95	40
Right FPl	Right Superior Frontal Gyrus	15,24,57	-5.59	143

^a^ Significant changes were only observed in schizophrenia patients<healthy comparison subjects, with a false discovery rate p<0.05, two-tailed, cluster size>30 voxels.

^b^ Voxel size was 3 × 3 × 3 mm^3^

FPl, lateral subreion of the frontal pole.

ROI, region of interest.

### Volumetric Atrophy of the FP Subregions in schizophrenia

**Fig 4:**
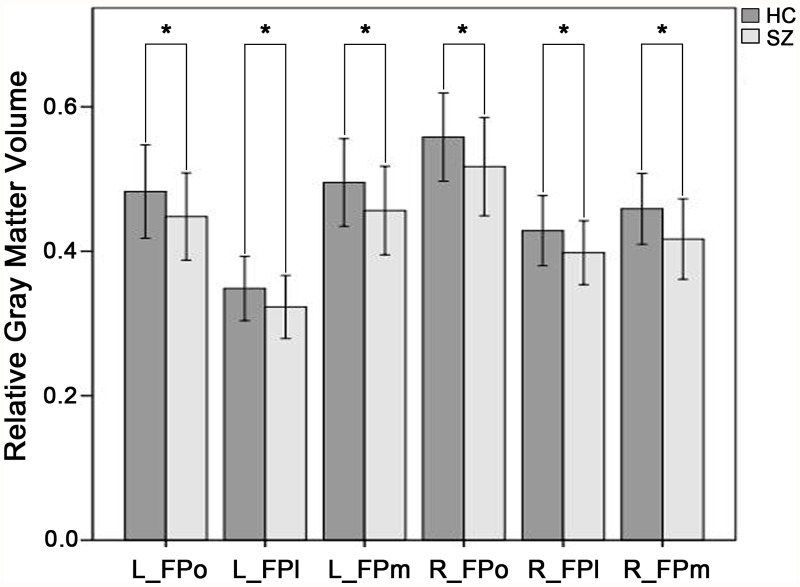
Comparisons of GMV of subregions of the frontal pole between schizophrenia patients and healthy controls. Y axis represents relative gray matter volume and X axis represents subregions of the frontal pole. Dark grey bar indicates healthy controls (HC) and light grey bar denotes schizophrenia patients (SZ). Star indicates p<0.001, Bonferroni corrected. FPo, orbital subregion of the frontal pole; FPm, medial subregion of the frontal pole; FPl, lateral subregion of the frontal pole; GMV, gray matter volume.

The schizophrenia patients showed significant decreased GMV in each of the subregions of the FP bilaterally (p<0.05, Bonferroni corrected) (Fig. 4).

### Validation of group difference in rsFC

Considering that ROIs extracted from the maximal probability maps may result in overlap across ROIs due to the processing of the normalization and smoothing, we also adopted another method (spheres with a radius of 6mm centered at the gravity of each FP subregion) to define these ROIs. We repeated our analyses and found that only the bilateral FPl demonstrated significant rsFC differences between schizophrenia patients and healthy controls (Fig. 3C and D). The locations of these clusters were largely overlapped with those derived from the method of the maximal probability map.

### The rsFC Differences of the FP Subregions after GMV Correction

To investigate whether the rsFC changes are associated with the underlying structural alterations, the rsFC analysis was repeated when the mean normalized GMV of each FPl of each subject was added as a covariate of no interest. All the clusters derived from rsFC analysis without GMV correction remained significant after GMV correction (Fig. 3E and F). These findings suggest that rsFC alterations of the bilateral FPl are relative independent characteristics in schizophrenia but not a result of volumetric atrophy.

### Correlations between rsFC of the FP subregions and clinical parameters

In patients with schizophrenia, we did not find any significant correlations (p<0.05) between rsFCs of the FPl with significant group differences and any of the clinical parameters, including the PANSS positive scores, PANSS negative scores, duration of illness, and current antipsychotic dosage in chlorpromazine equivalents (S1, S2 and S3 Table).

## Discussion

In the present study, we used a modified fMRI technique to investigate rsFC changes in the FP subregions in schizophrenia. We only found altered connectivity between the FPl and brain regions involved in cognitive processing in schizophrenia, suggesting the selective functional disconnection of the FP subregions in schizophrenia.

In our study, we used the SENSE-SPIRAL sequence instead of the conventional EPI sequence. The susceptibility-induced geometric distortions and signal loss in air/tissue interfaces are the main drawback of the EPI sequence [26–28]. However, the SENSE-SPIRAL sequence has been proved to be superior to the EPI sequence in terms of reducing sensitivity to motion, improving signal recovery and reducing geometric distortion [29]. The FP is often affected by susceptibility-induced artifacts in the EPI images [29]. In our study, the fMRI data acquired by SENSE-SPIRAL sequence exhibited less distortion in the FP and orbitofrontal regions compared with fMRI data acquired by EPI sequence. Consequently, fMRI data acquired by SENSE-SPIRAL sequence were used for rsFC analyses in the present study.

The human FP has dense connections with other higher-order association areas [3] and plays a crucial role in complex human cognition [6, 30]. As a reflection of functional segregation, we found that the FPo, FPm and FPl were implicated in three parallel neural networks that processing socio-emotional, self-referential, and cognitive information, respectively [2]. As a reflection of functional integration, we also found that the three FP subregions had several common rsFCs with a set of brain regions that are involved in cognitive processing, such as the MPFC, PCC/Pcu, TC and IPL. Most of these regions are activated together with the FP during a variety of cognitive tasks, such as the MPFC in prospecting the future [31], self-referential [6, 11, 32] and envisaging the perspectives of others [11, 33]; the IPL in exploratory decision and episodic memory retrieval [34, 35]; and the PCC/Pcu in episodic memory retrieval and self-related processing [36, 37]. In support of an integrative function of the FP, it has been shown to bias the priority of information from stimulus-oriented and stimulus-independent cognitive operations [30, 38].

The most important finding of this study is that the FPl was selectively impaired in schizophrenia. Specifically, schizophrenia patients exhibited reduced rsFCs between the FPl and the DLPFC, ACC, MPFC, TC, PCC/Pcu and IPL, all of which are involved in cognitive processing. The cognitive impairment is a core characteristic in schizophrenia [39]. Some authors even argue that schizophrenia should be considered as a cognitive illness because cognitive underperformance constitutes a (genetic) risk factor, precedes the onset of psychosis, continue to worsen after psychosis, and determines outcomes [40]. The FPl is mainly connected with the cognitive execution network (CEN), including the DLPFC and IPL, which are closely related to cognitive execution [41]. Both GMV atrophy and activation abnormalities in the DLPFC and IPL have been reported in schizophrenia [13, 42–44]. Consistent with our findings, functional disconnection between the FP and the IPL has also been reported in schizophrenia [45]. The ACC, a region crucial for cognitive control, has been frequently reported to show structural and functional abnormalities in schizophrenia [46–48]. The functional disconnection between the FPl and ACC may be associated with cognitive control impairment in schizophrenia. The MPFC and PCC/Pcu are important nodes of the default-mode network (DMN) and the connectivity and activation abnormalities have been found in schizophrenia [49–52]. The anterior temporal cortex is related to socio-emotional processes, and its dysfunction has also been reported in schizophrenia [53]. Taken all together, we found widespread functional disconnection between the FPl and the cognitive-related regions in schizophrenia, which may be related to cognitive impairments in this disorder.

Consistent with previous findings of GMV reduction in the FP [19–21], we further revealed that all FP subregions exhibited significant atrophy in schizophrenia. Several studies had suggested the link between the GMV and the functional brain activity [54, 55]. In this study, we did not find any effects of GMV atrophy of the FP subregions on the rsFC changes in schizophrenia. This finding suggests that rsFC alterations of the FPl are independent characteristics in schizophrenia but not a result of volumetric atrophy.

An important limitation in this study is that most of the patients were receiving different drug treatments. Although there is no correlation between drug dose and rsFC abnormalities in patients, we could not rule out the influence of antipsychotic medication on our results. A recent review indicated that antipsychotic medication may normalize the BOLD signal of the brain [56]. Future studies with first episode schizophrenic patients are required to eliminate the medication effects and confirm the findings of this study.

## Conclusion

In conclusion, to the best of knowledge, this is the first study to identify the resting-state functional connectivity alterations of the frontal pole in schizophrenia at the level of subregion using a more reliable fMRI technique. We found a selective functional disconnection between the lateral subregion of the frontal pole and brain regions involved in cognitive processing, which may improve our understanding of the functional disconnection in schizophrenia.

## Supporting Information

S1 Fig Altered resting-state functional connectivity (rsFC) of the right lateral subregions of the frontal pole in schizophrenia patients at a more liberal threshold. Between-group differences in the rsFC of the right lateral subregions of the FP demonstrate highly similar patterns with that of the left at a liberal uncorrected threshold of p<0.05. FP, frontal pole; FPl, lateral subregion; L, left, R, right.
(TIF)

S1 Table Correlations of functional connectivity of FPl subregions with PANSS positive or negative scores in schizophrenia patients. Brain regions indicate brain areas showed significant group differences in functional connectivity of FPl subregions. The *P* values are uncorrected and *r* denotes partial correlation coefficient FPl, lateral subregion of the frontal pole. PANSS indicates Positive and Negative Syndrome Scale. ROI, region of interest.
(DOCX)

S2 Table Correlations of functional connectivity of FPl subregions with current antipsychotic dosage in schizophrenia patients. Brain regions indicate brain areas showed significant group differences in functional connectivity of FPl subregions. The *P* values are uncorrected and *r* denotes partial correlation coefficient. FPl, lateral subregion of the frontal pole. PANSS indicates Positive and Negative Syndrome Scale. ROI, region of interest.
(DOCX)

S3 Table Correlations of functional connectivity of FPl subregions with duration of illness in schizophrenia patients. Brain regions indicate brain areas showed significant group differences in functional connectivity of FPl subregions. The *P* values are uncorrected and *r* denotes partial correlation coefficient. FPl, lateral subregion of the frontal pole. PANSS indicates Positive and Negative Syndrome Scale. ROI, region of interest.
(DOCX)
