# Supplementary figures and images for: The Selective Impairment of Resting-State Functional Connectivity of the Lateral Subregion of the Frontal Pole in Schizophrenia
Source: PLoS One. 2015 Mar 6;10(3):e0119176. doi: 10.1371/journal.pone.0119176 (PMC4352081; doi:10.1371/journal.pone.0119176)

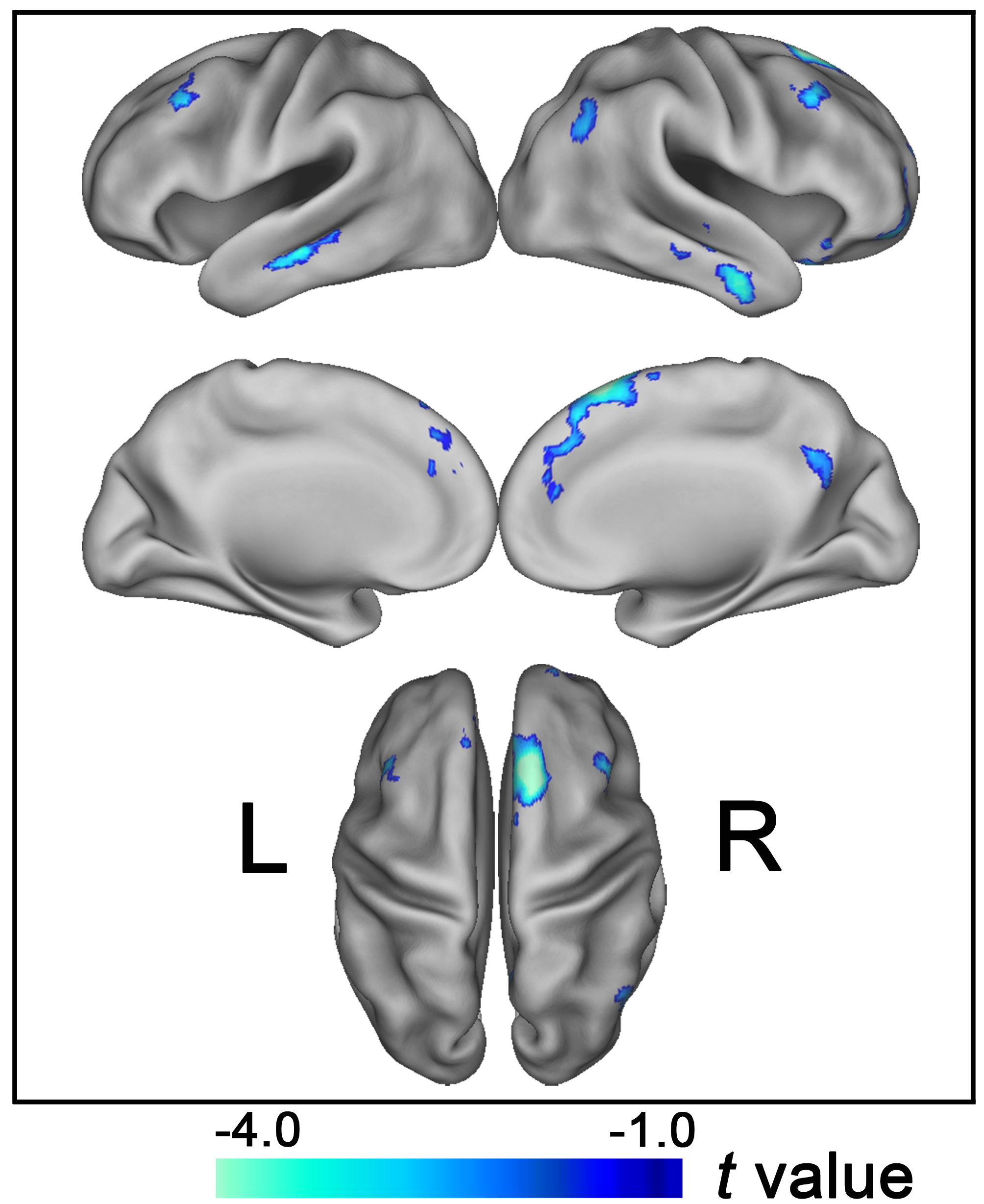

Supplement: S1 Fig — Between-group differences in the rsFC of the right lateral subregions of the FP demonstrate highly similar patterns with that of the left at a liberal uncorrected threshold of p<0.05. FP, frontal pole; FPl, lateral subregion; L, left, R, right. (TIF) [file pone.0119176.s001.tif]
