# Supplementary material for: The Selective Impairment of Resting-State Functional Connectivity of the Lateral Subregion of the Frontal Pole in Schizophrenia
Source: PLoS One. 2015 Mar 6;10(3):e0119176. doi: 10.1371/journal.pone.0119176 (PMC4352081; doi:10.1371/journal.pone.0119176)
Supplement: S1 Table — Brain regions indicate brain areas showed significant group differences in functional connectivity of FPl subregions. The P values are uncorrected and r denotes partial correlation coefficient FPl, lateral subregion of the frontal pole. PANSS indicates Positive and Negative Syndrome Scale. ROI, region of interest. (DOCX) [file pone.0119176.s002.docx]

**S2 Table. Correlations of functional connectivity of FPl subregions with PANSS positive or negative scores in schizophrenia patients.**

| **ROI** | **Brain regions** | **PANSS positive score** | | **PANSS negative score** | |
| --- | --- | --- | --- | --- | --- |
|  |  | *r* | *P* | *r* | *P* |
| **Left FPl** | Left middle temporal gyrus | 0.05 | 0.66 | -0.07 | 0.49 |
|  | Right middle temporal gyrus | -0.11 | 0.29 | -0.25 | 0.08 |
|  | Left anterior cingulate cortex | 0.07 | 0.49 | -0.11 | 0.32 |
|  | Left superior frontal gyrus | -0.05 | 0.65 | -0.18 | 0.09 |
|  | Left medial superior frontal gyrus | -0.01 | 0.92 | -0.16 | 0.14 |
|  | Left middle frontal gyrus | 0.02 | 0.87 | -0.09 | 0.42 |
|  | Left precuneus | 0.05 | 0.64 | -0.11 | 0.32 |
|  | Left angular gyrus | 0.13 | 0.22 | -0.10 | 0.37 |
|  | Right angular gyrus | -0.14 | 0.19 | -0.11 | 0.29 |
| **Right FPl** | Right superior frontal gyrus | -0.08 | 0.48 | -0.10 | 0.34 |
