# Supplementary material for: The Selective Impairment of Resting-State Functional Connectivity of the Lateral Subregion of the Frontal Pole in Schizophrenia
Source: PLoS One. 2015 Mar 6;10(3):e0119176. doi: 10.1371/journal.pone.0119176 (PMC4352081; doi:10.1371/journal.pone.0119176)
Supplement: S2 Table — Brain regions indicate brain areas showed significant group differences in functional connectivity of FPl subregions. The P values are uncorrected and r denotes partial correlation coefficient. FPl, lateral subregion of the frontal pole. PANSS indicates Positive and Negative Syndrome Scale. ROI, region of interest. (DOCX) [file pone.0119176.s003.docx]

**S3 Table.** **Correlations of functional connectivity of FPl subregions with current antipsychotic dosage in schizophrenia patients.**

| **ROI** | **Brain regions** | **Current antipsychotic dosage (chlorpromazine equivalents)** | |
| --- | --- | --- | --- |
|  |  | *r* | *P* |
| **Left FPl** | Left middle temporal gyrus | 0.05 | 0.62 |
|  | Right middle temporal gyrus | -0.01 | 0.95 |
|  | Left Anterior cingulate cortex | 0.07 | 0.49 |
|  | Left superior frontal gyrus | 0.05 | 0.67 |
|  | Left medial superior frontal gyrus | 0.04 | 0.72 |
|  | Left middle frontal gyrus | 0.14 | 0.19 |
|  | Left precuneus | 0.13 | 0.22 |
|  | Left angular gyrus | 0.03 | 0.78 |
|  | Right angular gyrus | -0.09 | 0.42 |
| **Right FPl** | Right superior frontal gyrus | -0.13 | 0.22 |
