# Supplementary material for: The Selective Impairment of Resting-State Functional Connectivity of the Lateral Subregion of the Frontal Pole in Schizophrenia
Source: PLoS One. 2015 Mar 6;10(3):e0119176. doi: 10.1371/journal.pone.0119176 (PMC4352081; doi:10.1371/journal.pone.0119176)
Supplement: S3 Table — Brain regions indicate brain areas showed significant group differences in functional connectivity of FPl subregions. The P values are uncorrected and r denotes partial correlation coefficient. FPl, lateral subregion of the frontal pole. PANSS indicates Positive and Negative Syndrome Scale. ROI, region of interest. (DOCX) [file pone.0119176.s004.docx]

**S4 Table. Correlations of functional connectivity of FPl subregions with duration of illness in schizophrenia patients.**

| **ROI** | **Brain regions** | **Duration of illness** | |
| --- | --- | --- | --- |
|  |  | *r* | *P* |
| **Left FPl** | Left middle temporal gyrus | 0.02 | 0.84 |
|  | Right middle temporal gyrus | 0.01 | 0.93 |
|  | Left Anterior cingulate cortex | -0.21 | 0.05 |
|  | Left superior frontal gyrus | -0.08 | 0.44 |
|  | Left medial superior frontal gyrus | -0.02 | 0.84 |
|  | Left middle frontal gyrus | -0.05 | 0.68 |
|  | Left precuneus | 0.11 | 0.33 |
|  | Left angular gyrus | -0.07 | 0.51 |
|  | Right Angular Gyrus | -0.12 | 0.25 |
| **Right FPl** | Right Superior Frontal Gyrus | -0.11 | 0.29 |
